# Feasibility, technique and accuracy of ultrasound-guided transurethral injections into the urinary sphincter of female cadavers: proof of concept

**DOI:** 10.1186/s12894-020-00719-x

**Published:** 2020-10-23

**Authors:** Florian A. Schmid, Dominic Gascho, Niklaus Zoelch, Jenny A. Prange, Giovanni Colacicco, Daniel Eberli

**Affiliations:** 1 Department of Urology, University Hospital of Zurich, University of Zurich, Frauenklinikstrasse 10, 8091 Zurich, Switzerland; 2 Institute of Forensic Medicine, University of Zurich, Zurich, Switzerland; 3 Institute of Anatomy, University of Zurich, Zurich, Switzerland

**Keywords:** Transurethral injection, Urinary sphincter, Ultrasound-guided, MRI, Female

## Abstract

**Background:**

The injection of muscle precursor cells (MPC) into the external urinary sphincter muscle (EUS) is a promising therapeutic option for regenerative treatment of stress urinary incontinence (SUI). The objective of the present project was to conduct a pre-clinical trial to investigate the feasibility and accuracy of ultrasound (US) guided, transurethral injections into the EUS of female cadavers.

**Methods:**

This is a prospective, anatomical, interventional and radiological cadaveric laboratory investigation. Two urologists performed transurethral US-guided injections to deliver nano-iron particles into the EUS. The intervention was performed in three unfixed, fresh female cadavers. Each cadaver received MRI before and CT as well as MRI of the pelvis after the injections.

**Results:**

The precision and accumulation of nano-iron particles in the EUS was compared using a rating scale to evaluate left versus right and anterior versus posterior distribution in axial and sagittal orientation with US, MRI and CT. The accuracy of our US-guided injections into the anterior target region yielded 4 points on the rating scale. Adequate precision and accumulation of particles in the left versus right EUS were also demonstrated (3 vs. 3.33 points, respectively). Signal intensity in MRI revealed a mean ratio of 0.33 before and after injection. CT scans showed no relevant artefacts impairing the assessment.

**Conclusion:**

US-guided, transurethral injection into the EUS is feasible and imaging reveals a precise accumulation in the target region. Our method provides an appropriate approach to deliver MPC in the EUS muscle for a regenerative treatment of SUI in the near future.

## Background

Stress Urinary Incontinence (SUI) is a disease affecting almost 200 million people worldwide and is twice as common in women as in men [1]. The quality of life in these patients is markedly decreased due to reduced activity, unpleasant sensation and odor as well as recurrent urinary tract infections caused by wet pads [2, 3]. Several therapeutic options exist in the treatment of SUI: Besides physiotherapy of the lower pelvis, various medical, mechanical and surgical approaches can be discussed with the patients [4–8]. However, all of these therapies have limitations and new solutions with long-term effects are needed. The regenerative approach, administering muscle precursor cells (MPC), is a promising new strategy to strengthen the external urinary sphincter muscle (EUS) in a sustainable manner in patients with SUI [9–11]. Pursuing the ultimate goal of treating the cause rather than the symptom was already convincing in the animal model, and the translation of this therapy into humans demonstrated first promising results in the past [12, 13]. However, particularly the precise deliverance of MPCs into the target region is a challenging endeavor. Stem cells are highly influenced by the microenvironment and therefore an accurate injection into the external EUS is required. The aim of the present study was to prove the feasibility and precision of a new ultrasound (US) guided transurethral injection technique into the EUS.

## Methods

In this prospective, anatomical, interventional and radiological cadaveric laboratory investigation, we included three unfixed, fresh female cadavers for research purposes between May 2019 and January 2020. As initial baseline investigation, a radiological technologist performed magnetic resonance imaging (MRI) of the lower pelvis at the Institute of Forensic Medicine shortly after arrival of the cadaver. Before, the cadaveric bodies were stored at 4 °C for a maximum of 72 h. After imaging, we transported the bodies to the “Skills Laboratory” of the Institute of Anatomy and put them into lithotomy position. The lower pelvis was investigated by initial vaginal US carried out by two urologists, performing a 3D block of the lower pelvis. This allowed the identification of important anatomical landmarks and the location of the EUS in mid-urethral position as a hypoechoic structure. The next step was the insertion of a transurethral indwelling bladder catheter (12 French) and the filling of the catheter balloon with 10 ml (ml) of saline solution. Further, a urethral therapy (stainless steel) shaft was attached to the catheter outside of the urethral meatus. After filling of the bladder with 100 ml of saline solution, we attached a Kochers’ clamp at the rear of the catheter to prevent outflow. Further, this assured fixation of the catheter balloon at the bladder neck as an anatomical reference structure. We then reassured a good position of the vaginal US probe and double-checked image quality. Thereafter, the urethral therapy shaft was inserted into the urethral meatus in Seldinger technique along the transurethral catheter until mid-urethral position and then linked to the vaginal ultrasound probe. This led to a signal loss in the distal part of the US image. After bringing in a puncture cannula through the therapy shaft, the tip of the needle was identified in the target region at 12 o’clock position. The needle was then adapted to a syringe with a cooled solution of 4 ml nano-iron particles (Endorem®, Guerbet AG, dextran-coated SPIO nanoparticles, size distribution 120–180 nm, iron-content 11.2 mg/ml), that was transported to the facility of the Anatomical Institute in a refrigerated box. For the injection in cadaver 1, the nano iron-particles were used in the above-mentioned, original dosage. Due to a massive extinction signal in cadaver 1 (Additional file 1: Appendix, Figs. 4 and 5), we diluted the particles in a 1:100 manner with saline solution for cadavers 2–3 (Additional file 1: Appendix, Figs. 4 and 5). Further, the nano-iron particles were mixed with a collagen solution. Collagen changes its viscosity with increasing temperature and turns into a jelly composition. Therefore, an out- or backflow of the injection mixture through the puncture canal can be minimized in living humans. However, because the cadavers had to be stored in the fridge at 4 °C, this effect was only minimal. We started with the first injection at the 12 o’clock position and changed the angle gradually to the right or to the left for each of the subsequent injections. It was our intention to focus mainly on anterior injection, since the EUS has an inverse “horseshoe” configuration. At the end of the procedure, all of the instruments were removed. Back at the Institute of Forensic Medicine, each of the female corpses received a second MRI of the pelvis in order to compare the acquired images to those before the injection. Further, a computer tomography (CT) examination was performed to exclude artefacts in the region of interest, such as the presence of air bubbles caused by the injection. Air bubbles would alter the signal-intensity (SI) in MRI, whereas the quality of evaluation would be limited.

### MRI and CT scanning protocol

The MRI examination was performed using a 3 T MRI unit (Achieva 3.0 TX, Philips Medical System, Best, The Netherlands) and a 16 elements phased-array SENSE XL Torso coil. The scan protocol included a T1-weighted turbo-spin-echo (TSE) sequence (orientation: transversal; TR: 578 ms; TE: 6.8 ms; acquisition voxel size [mm]: 0.7 × 0.7 × 2.5), T2-weighted TSE sequences (orientation: transversal/coronal/sagittal; TR: 3765/3464/1958 ms; TE: 60 ms; acquisition voxel size [mm]: 0.8 × 0.8 × 2.5), and T2*-(susceptibility-) weighted fast-field-echo (FFE) sequences (orientation: transversal/coronal; TR: 1117 ms; TE: 16 ms; acquisition voxel size [mm]: 0.9 × 1.2 × 2.5). The same MRI scanning protocol was used before and after the injection of the nano-iron particles.

The CT scans were performed using a 128-slice CT (SOMATOM Definition Flash, Siemens Healthcare, Forchheim, Germany). First, a CT scan over the entire pelvis was performed. The scan parameters were 120 kVp, 800 mAs, and a pitch of 0.35. The raw data were reconstructed using a slice thickness of 0.6 with a hard kernel (B60) and a soft kernel (B30). Second, a high-resolution CT over the region of the sphincter was performed using 120 kVp, 330 mAs, and a pitch of 0.35. Reconstructions were calculated using small field-of-view (50 × 50 mm; pixel spacing (in-plane voxel): 0.098 mm) with a slice thickness of 0.4 mm and a hard kernel (U70) [14].

### Evaluation of imaging

**Fig. 1:**
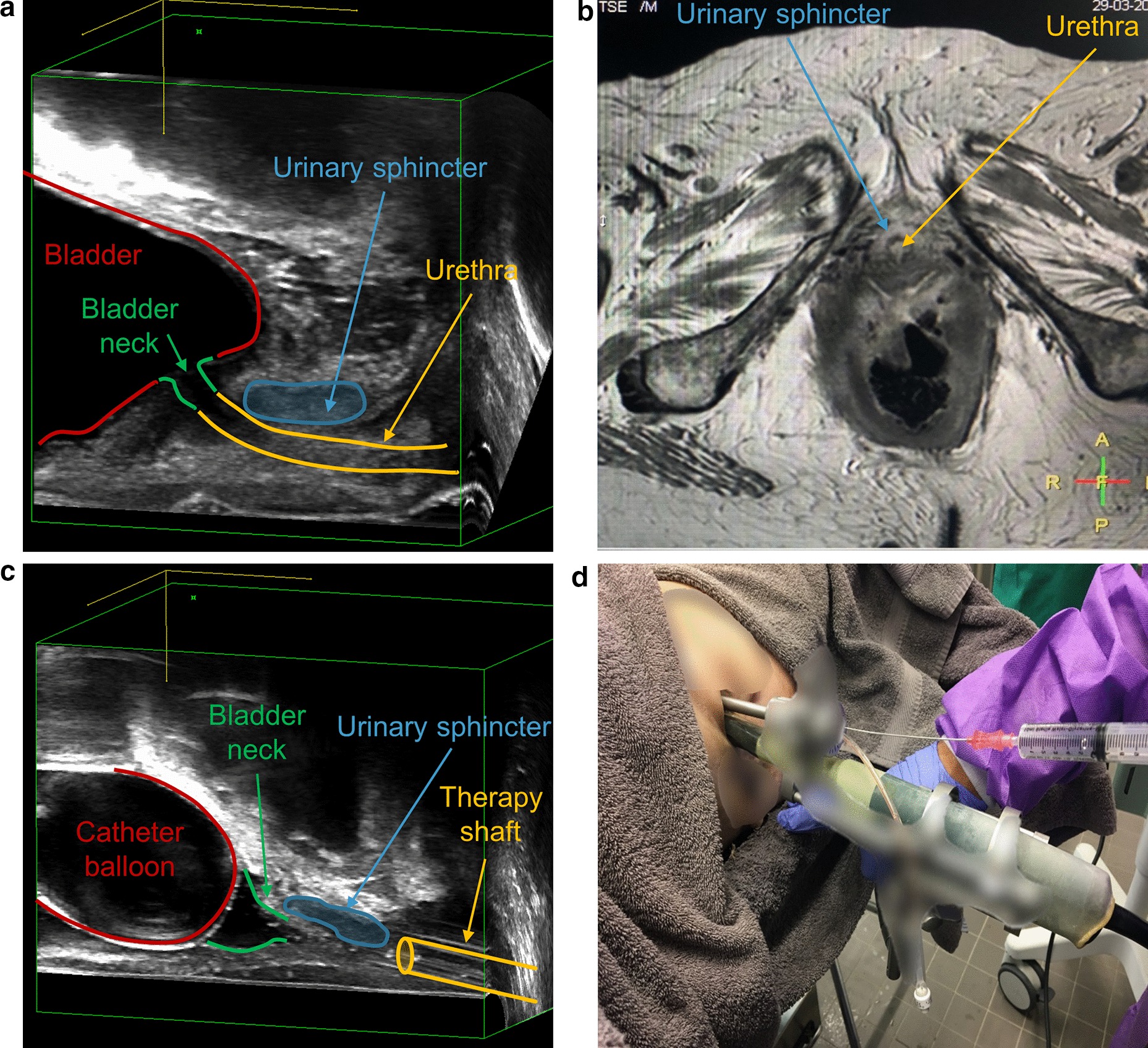
**a** Anatomy of the lower female pelvis in 3D US with transversal view and **b** MRI T2 sequence in axial view. **c** Setting before transurethral injection of nano-iron particles. Extracorporeal setup, where vaginal US probe and urethral therapy shaft are linked together. **d** Needle puncture and injection are performed through the therapy shaft

**Fig. 2:**
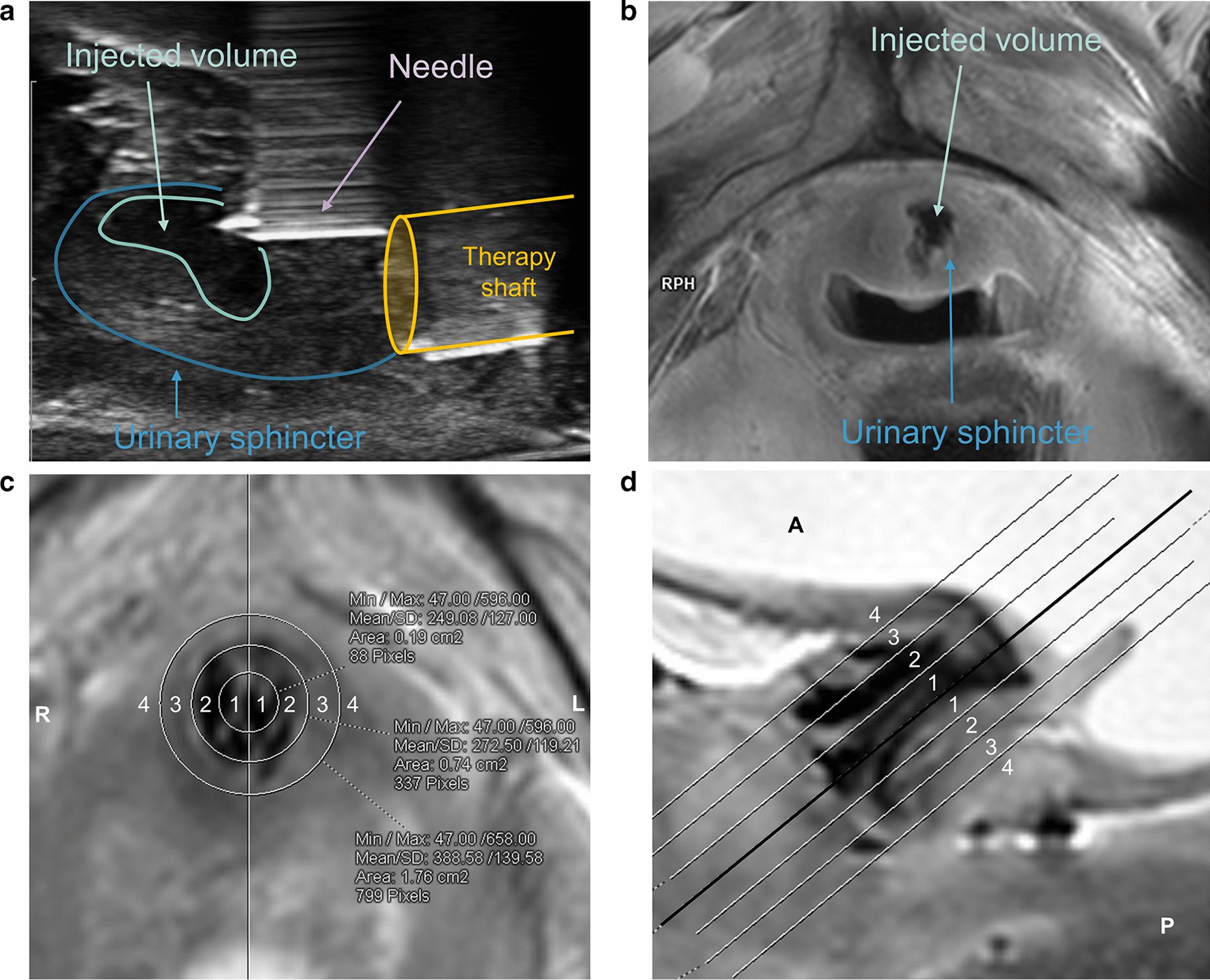
**a** US image in sagittal orientation during transurethral injection. **b** MRI image in axial orientation after injection of nano-iron particles. **c**, **d** Scoring of injection defined on a rating scale from 1 to 4 points according to extinction signal of injected iron nano-particles in MRI images. **c** Axial orientation with division into right and left, **d** sagittal orientation with division into anterior and posterior. Diameter and distances in (**a**) and (**b**), respectively: 1 = 5 mm, 2 = 10 mm, 3 = 15 mm, 4 > 15 mm

**Fig. 3:**
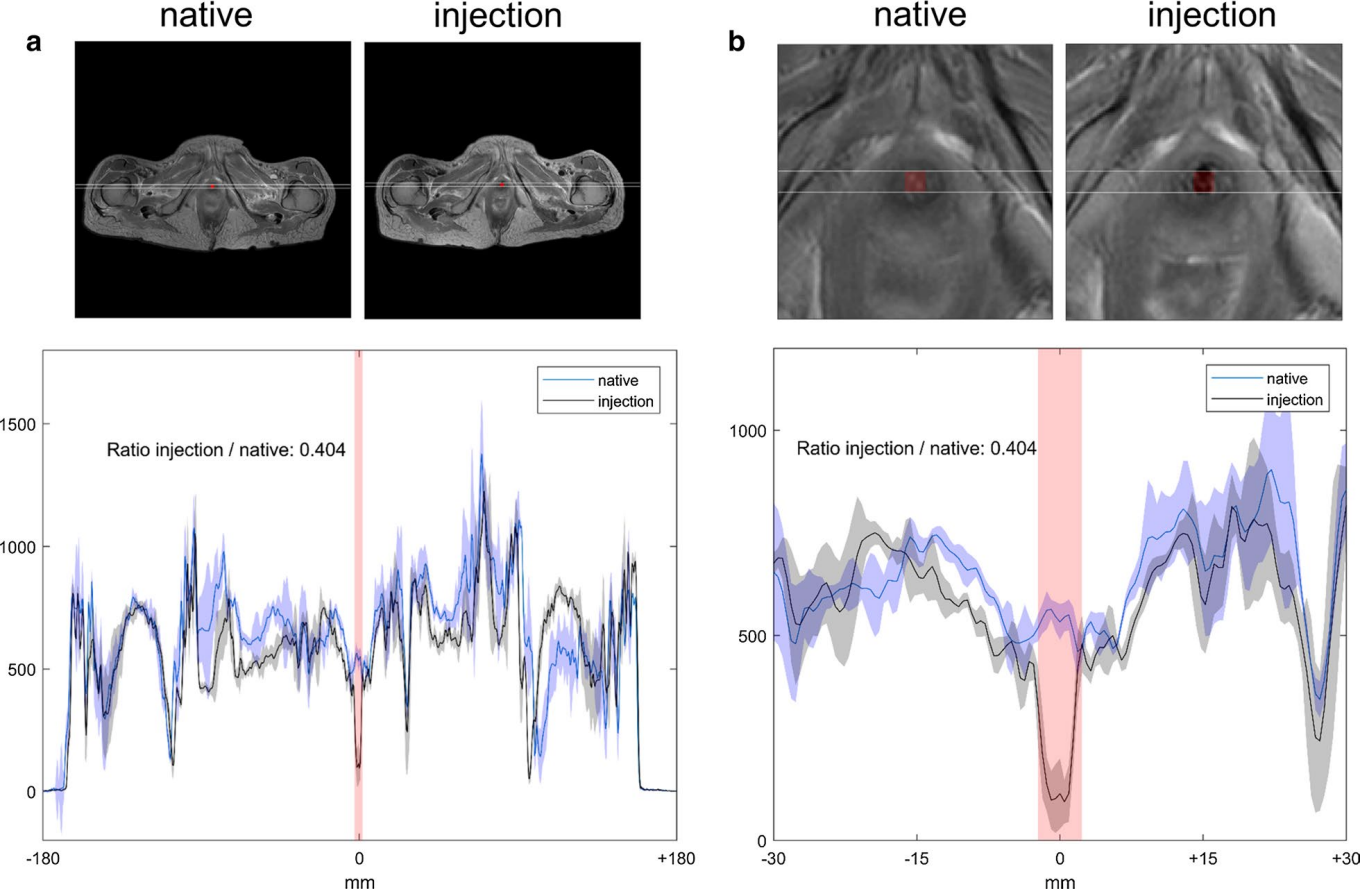
Evaluation of the ratio of signal intensity in the EUS muscle in MRI. Measured differences due to extinction before and after injection of nano-iron particles in Cadaver 3. The red area marks the EUS in all parts of the figure. **a** Overall, **b** zoom

Baseline MRI and an US 3D-block of the lower pelvis assessed the status quo and the exact anatomy before injection (Fig. 1a, b). US was then used as guidance and documentation of the whole injection procedure (Figs. 1c, d, 2a). The MRI and the additional CT scan after the injections were performed for the analysis of injection precision (Fig. 2b–d). With three different imaging modalities (US, MRI and CT), we were able to compare injection accuracy in an objective and qualitative way. We rated the precision of our injections according to a rating scale from 1–4 points: 1 = center (diameter/distance = 5 mm), 2 = inner ring/line (diameter/distance = 10 mm), 3 = middle ring/line (diameter/distance = 15 mm), 4 = outer ring/line (diameter/distance > 15 mm). For the evaluation, images were divided into left/right in axial as well as anterior/posterior in sagittal orientation (Fig. 2c, d). With this scoring system, the accuracy of injections through imaging was evaluated by the consensus of two independent raters (radiological technologist and urologist). The cumulative extinction signal in both orientations (sagittal and axial) was calculated as 3D-volumes. Additionally, we evaluated signal-intensity changes in MRI: According to signal-intensity curves (Fig. 3a, b, Additional file 1: Appendix Figs. 6 and 7), the ratio before and after injection of diluted nano-iron particles was calculated for the EUS region. To do so, standard protocols were used.

## Results

In each of the cadavers, all three imaging modalities—US, MRI and CT—were applied. In MRI, we have qualitatively evaluated the sharpness and details of the images for the inspection and extension of small muscle structures, such as the EUS muscle. Image quality was evenly distributed among all of the cadavers, except for artefacts in cadaver 2 due to bilateral hip prosthesis. However, these artifacts did not impede measurements on the rating sale; thus, this cadaver was not excluded. Further, the fixation of an US-probe to the therapy shaft and the subsequent imaging quality was of relevance and did not differ among cadavers. Additionally, it was important to evaluate the impact of ventral artefacts after inserting the urethral therapy shaft (metal composition) and to assess whether the needle could still be tracked precisely, which was the case in all three interventions. As described in the methods section, our injectable solution of nano-iron particles and collagen needed to be diluted with saline after the first procedure, due to strong extinction signals in cadaver 1 (Additional file 1: Appendix, Figs. 4 and 5). However, since the center of the extinction signal was in the right anatomical location, cadaver 1 was not excluded from the analysis. In total, the MRI revealed the location of our injections precisely at intended mid-urethral and anterior position (Additional file 1: Appendix, Figs. 4 and 5). CT scans additionally assured the absence of air bubbles in the area of injection (Additional file 1: Appendix, Figs. 4 and 5). The handling of the needle, assuring the correct depth of puncture and the equal distribution of the 4 ml solution through all the intended injections spots were feasible at all times. The assessment of injection location revealed a maximum score of 4 points on the rating scale in anterior orientation and comparable results regarding division of the injection volume were obtained left vs. right (3 vs. 3.33 points). The lowest amount was seen in the posterior region with a mean rating score of 2.67 points. The mean injection volume calculated by the MRI was 1.75 cm^3^ (Table 1). The measurement of the MRI extinction signal revealed a signal-intensity (SI) curve with a consistent ratio of less than a half (mean SI-ratio = 0.33) before and after injection of nano-iron particles into the EUS (reference SI: air ≈ 0, purefat ≈ 1500) (Table 1).

**Table 1:** Scores per cadaver on the rating scale according to extinction signal in MRI image in axial (left, right) and sagittal (anterior, posterior) orientation

Cadaver	Axial left	Axial right	Sagittal anterior	Sagittal posterior	Overall mean	Radius (mm)	Area (mm^2^)	Volume measured in MR (cm^3^)	SI-ratio
1	4	4	4	4	4	2.5	19.6	3.82	0.055
2	2	4	4	1	2.75	5.0	78.5	0.32	0.526
3	3	2	4	3	3	7.5	176.7	1.11	0.404
Column mean	3	3.33	4	2.67	3.25	5	91.60	1.75	0.33

Measured radius, area and volume of injection. Signal-intensity ratio before and after injection in the EUS

*SI* signal-intensity, *MR* magnetic resonance

## Discussion

In the present study, we were able to prove that our technique for transurethral injections into the EUS under US guidance is feasible and yields good precision as seen in subsequent imaging proof. The signal decrease in pelvis MRI demonstrates a mean ratio of 0.33 when comparing intensities before and after the injection of nano-iron particles into the EUS. The indication for the use of nano-iron particles (SPIO = superparamagnetic iron oxide) in clinical MRI became broad over the last years, but was initially intended for the enhanced identification of focal malignant liver lesions [15]. For our purpose of a local injection, the signal alterations due to enhanced susceptibility in T2*-sequences in MRI was ideal and additionally not visible in CT scans. Our investigation showed that the anatomical reference in T2*-weighted imaging shows adequate mid-urethral accumulation in the target region (EUS).

Substantial research has looked into the feasibility, adverse effects and outcome of different interventions to treat SUI in women. Recently, a Cochrane systematic review compared different urethral injection therapies and concluded that the current evidence remains insufficient to guide practice [16]. They reported a similar outcome of periurethral versus transurethral outcomes regarding symptoms of SUI, whereas early complications tended to be higher in the periurethral group. The cost-effectiveness of urethral bulking agents compared to sling surgery was doubted during a longer follow-up period of 15 months. Burdzinska and colleagues compared the accuracy between the transurethral and periurethral route of intrasphincteric injections of autologous cellular suspensions in goats [17]. Their investigation revealed that 2/3 of all injections could be located in the urethral wall, while less than 1/5 were found inside EUS muscle structures. The main difference to our investigation was the fact that transurethral injections were performed under endoscopic but no US guidance, which could be an explanation for the lower accuracy. American researches evaluated transurethral injections of bulking agents (Macroplastique^®^) by 3D endo-vaginal US to describe location and distribution after the intervention [18]. By using US, they reported good precision at 3 and 9 o’clock position of the EUS, but highly variable distances from the urethro-vesical junction. Blaganje et al. demonstrated, that transurethral injections of autologous myoblasts through US guidance are feasible and reveals improvement of symptoms in combination with functional electrical stimulation in more than 75% of female patients suffering from SUI [19]. Interestingly, Aicher and his group recently published a needle-free technique using a novel waterjet injection to deposit mesenchymal stromal cells into the EUS of fresh porcine urethrae [20]. The yield of viable cells was significantly higher than by injection through a William’s needle while assuring similar accuracy. Therefore, this novel approach could be a promising technique to deposit viable cells into the EUS in the future.

A limitation of this proof of concept is the post-mortem character of our investigation. The injection and imaging in female cadavers may differ from in-vivo procedures and therefore results need to be interpreted with caution. Further, the use of nano-iron particles for MPC tracking was considered inappropriate for living humans, since macrophages tend to uptake and evacuate MPCs previously stained with iron oxide [21]. In addition, the two independent raters (radiological technologist and urologist) were not blinded to the treatment method, which could potentially induce assessor bias. However, the evaluation with the rating scale was performed in reference to the urethra as an anatomical structure and was the same in all three cadavers. Therefore, and according to our imaging series, the approach and the technique may be reliably used for the accurate deposition of injectables through the transurethral route under US guidance.

## Conclusion

Our US-guided injection technique for EUS application is feasible and shows an adequate as well as precise accumulation of nano-iron particles in the desired target region, as confirmed by MRI. It may be applied as a simple approach to accurately deliver MPCs in the EUS for the regenerative treatment of female SUI in the near future.

## Supplementary information

**Additional file 1:** Appendix with presented Figs. 4–7 as supplement to Figs. in the text.

## Data Availability

This study includes data and imaging from dead human bodies. For reasons of reverence, datasets used and/or analysed during the current study are available from the corresponding author on reasonable request only.
