# Supplementary material for: Feasibility, technique and accuracy of ultrasound-guided transurethral injections into the urinary sphincter of female cadavers: proof of concept
Source: BMC Urol. 2020 Oct 23;20:167. doi: 10.1186/s12894-020-00719-x (PMC7583166; doi:10.1186/s12894-020-00719-x)

**Appendix A**


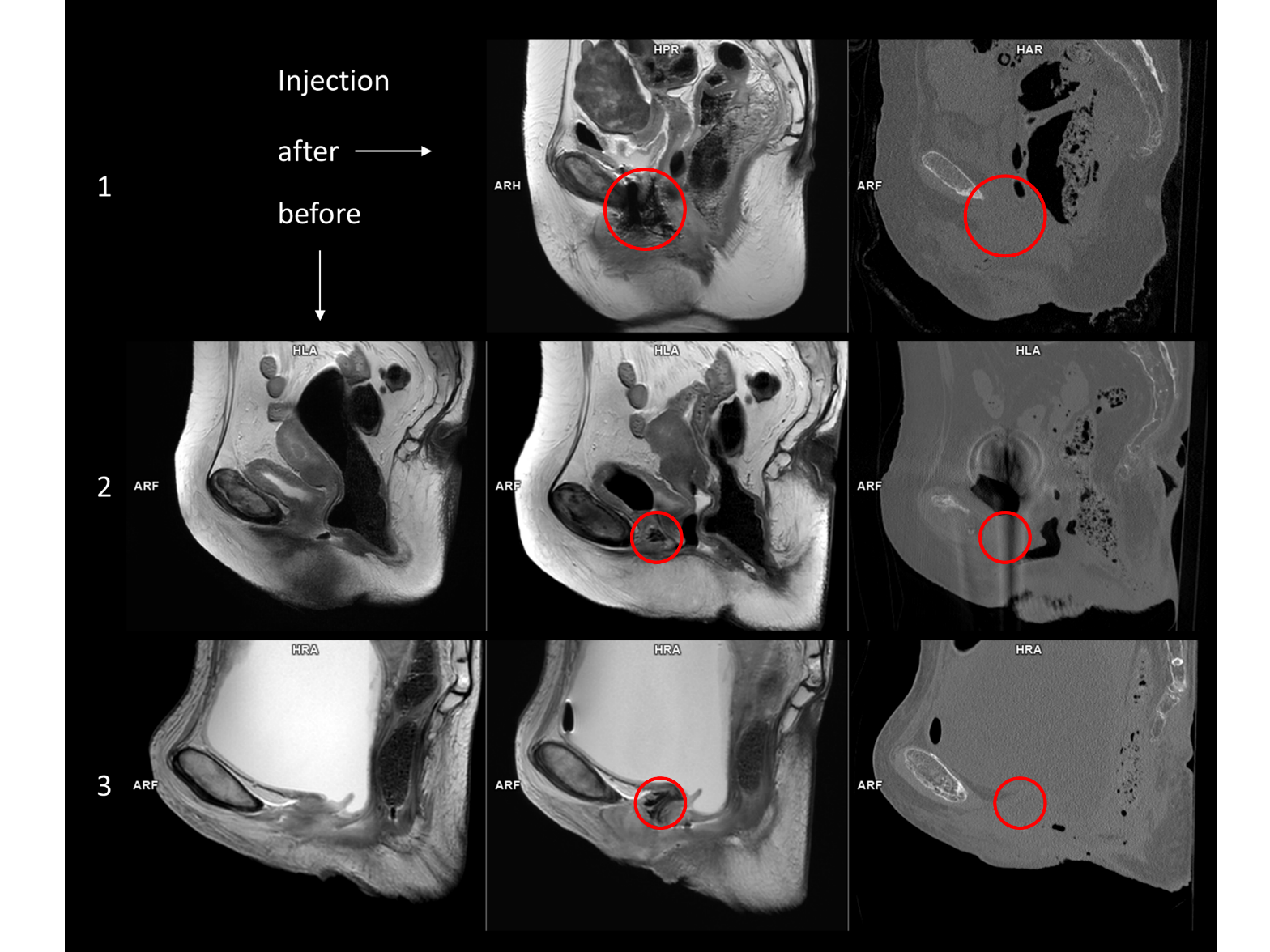
*Figure A1:* Visual comparison before (first column) and after injection (second/third column) in sagittal MRI (first/second column) and CT (third column) in Cadaver 1-3. Although cadaver 2 had a hip prosthesis (right column, CT image), the metal artifacts did not impede the rating scale MRI.


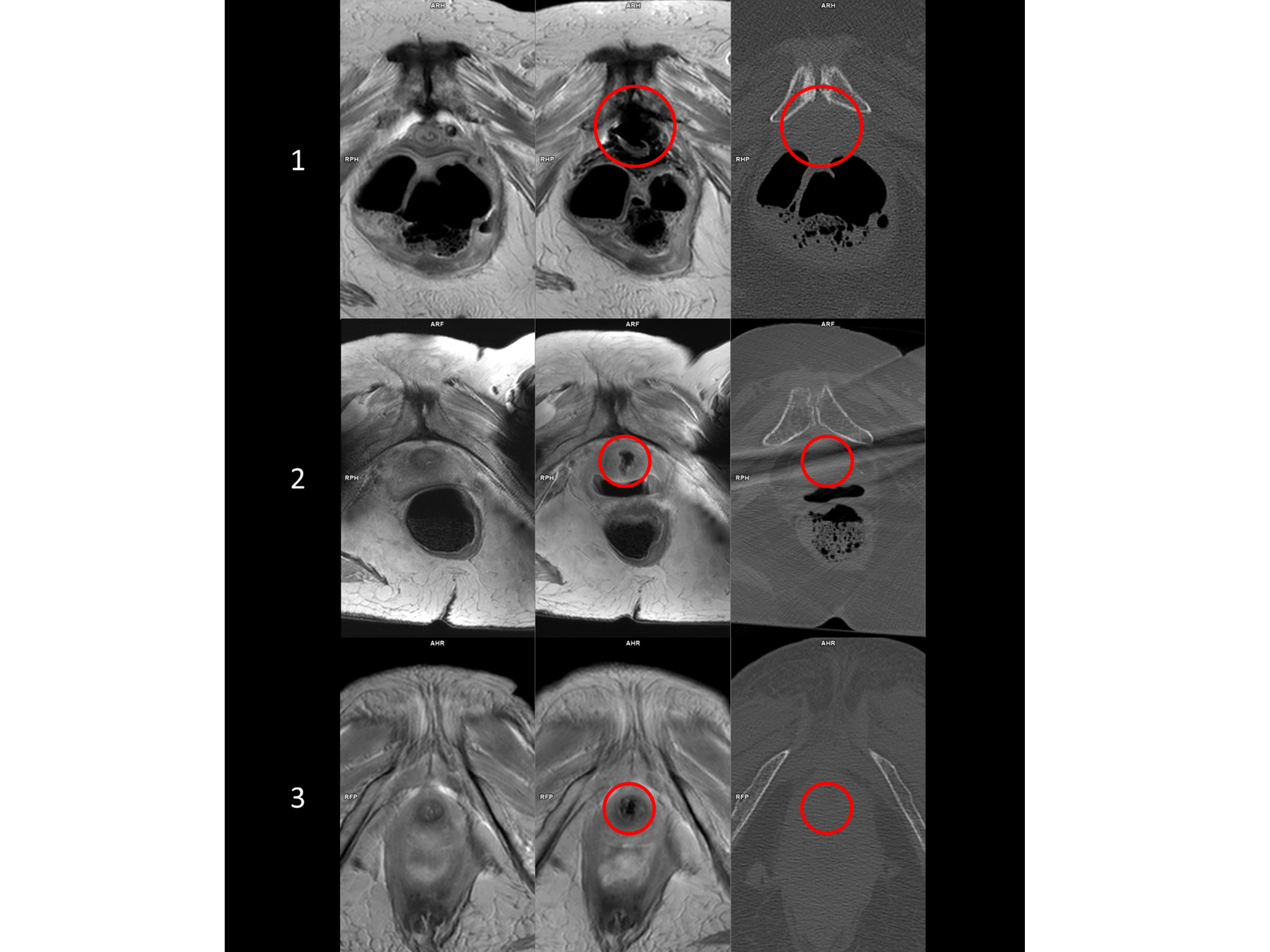
*Figure A2:* Visual comparison before (first column) and after injection (second/third column) in axial MRI (first/second column) and CT (third column) in Cadaver 1-3. The metal artifacts of a hip prosthesis in cadaver 2 (dark stripe on the CT image) did not impede the rating scale on MRI.

**Appendix B**

*Figure B1:* Evaluation of the ratio of signal intensity in the EUS muscle in MRI. Measured differences due to extinction before and after injection of nano-iron particles in Cadaver 1. The red area marks the EUS in all parts of the figure. A: Overall. B: Zoom.


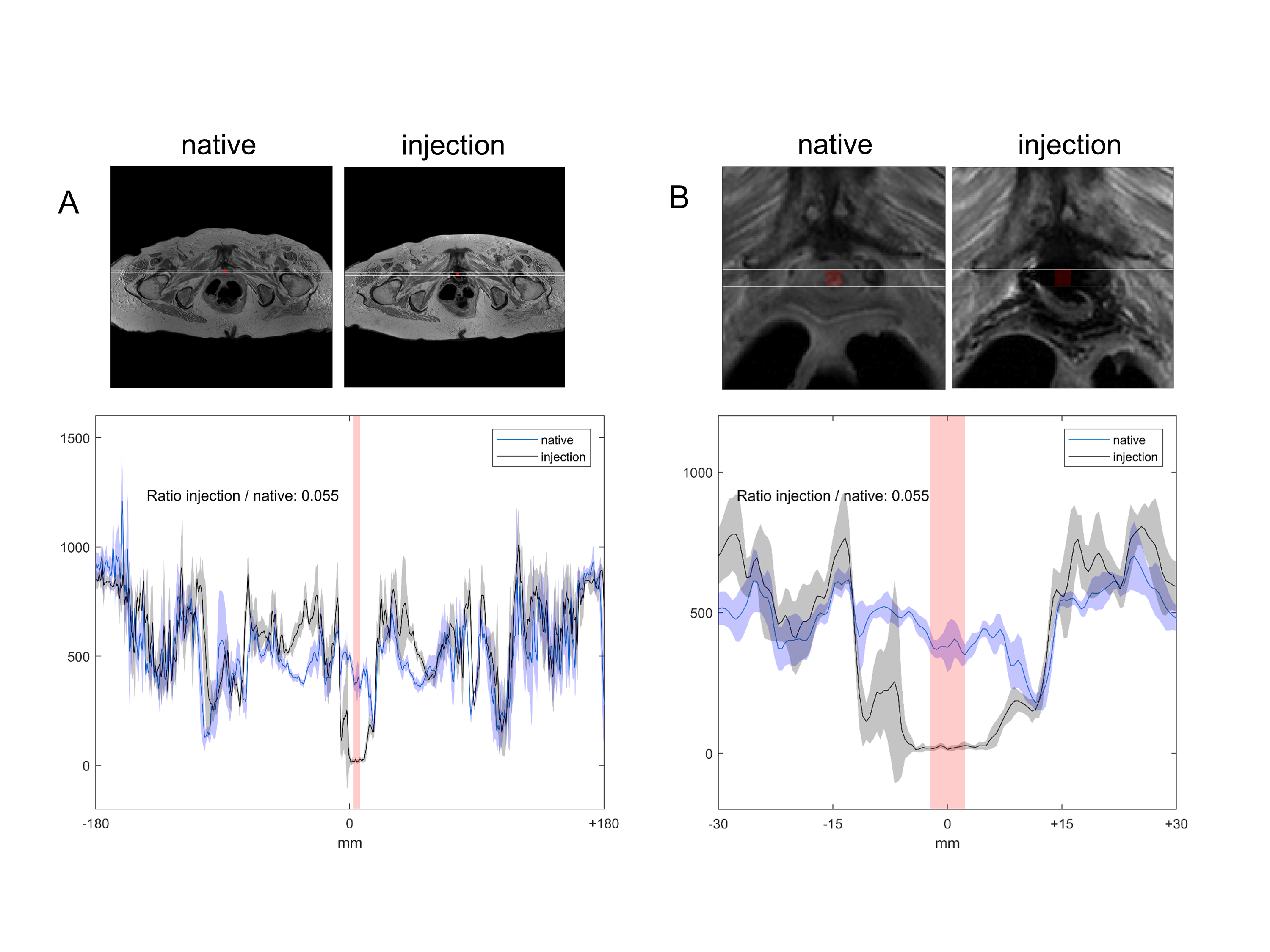


*Figure B2:* Evaluation of the ratio of signal intensity in the EUS muscle in MRI. Measured differences due to extinction before and after injection of nano-iron particles in Cadaver 2. The red area marks the EUS in all parts of the figure. A: Overall. B: Zoom. Artefacts in A are explained due to presence of bilateral hip prosthesis.


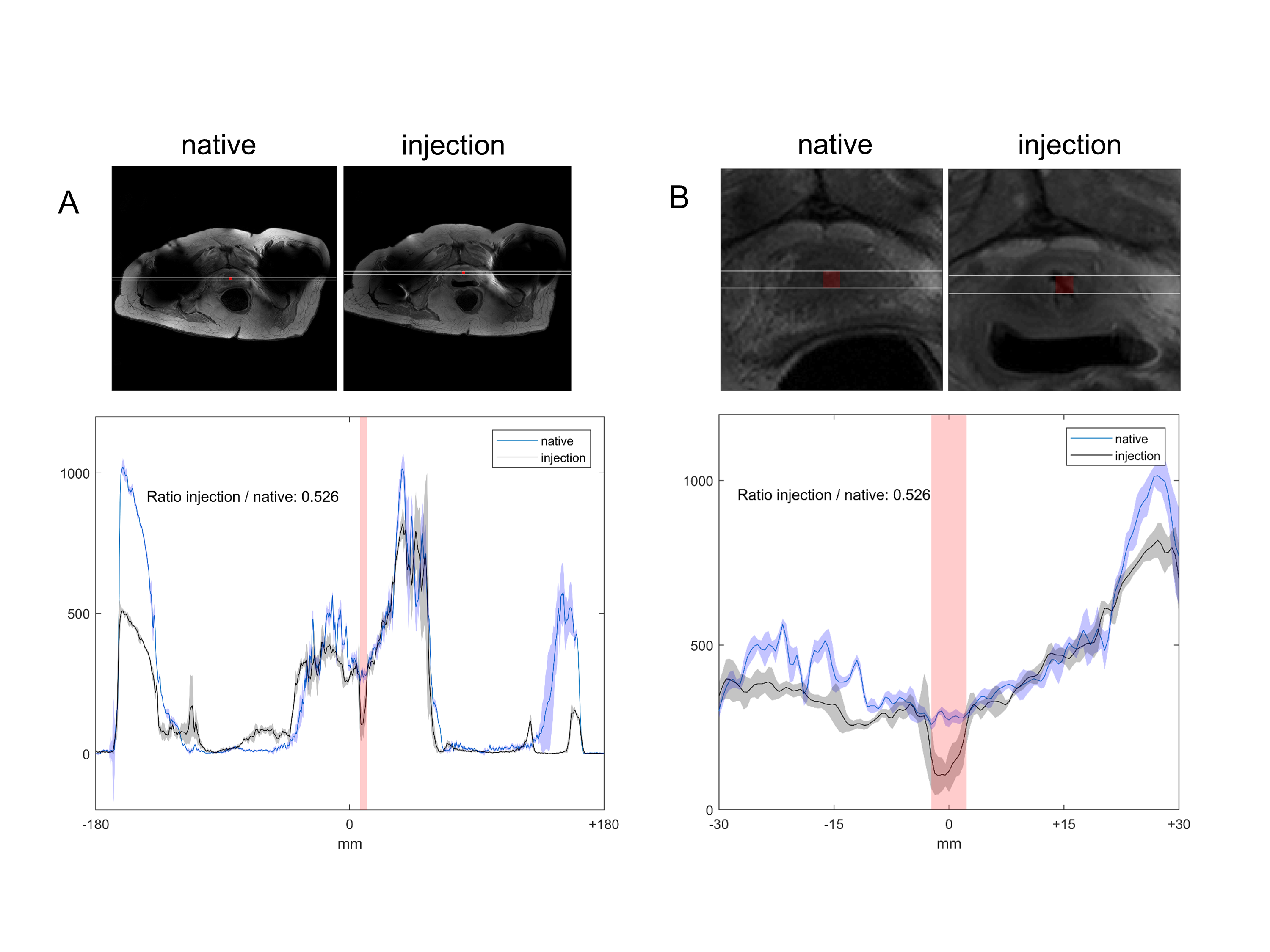

Supplement: Supplementary file 1 — Additional file 1: Appendix with presented Figs. 4–7 as supplement to Figs. in the text. [file 12894_2020_719_MOESM1_ESM.docx]
